# Evaluation of Brain Tortuosity Measurement for the Automatic Multimodal Classification of Subjects with Alzheimer's Disease

**DOI:** 10.1155/2020/4041832

**Published:** 2020-01-29

**Authors:** Eduardo Barbará-Morales, Jorge Pérez-González, Karla C. Rojas-Saavedra, Verónica Medina-Bañuelos

**Affiliations:** ^1^Neuroimaging Laboratory (LINI), Electrical Engineering Department, Universidad Autónoma Metropolitana-Iztapalapa (UAM-I), Mexico City, Mexico; ^2^Instituto de Investigaciones en Matemáticas Aplicadas y Sistemas (IIMAS), Sede Mérida, Universidad Nacional Autónoma de México (UNAM), Mexico City, Mexico; ^3^Universidad del Valle de México (UVM) Campus Sur, Mexico City, Mexico

## Abstract

The 3D tortuosity determined in several brain areas is proposed as a new morphological biomarker (BM) to be considered in early detection of Alzheimer's disease (AD). It is measured using the sum of angles method and it has proven to be sensitive to anatomical changes that appear in gray and white matter and temporal and parietal lobes during mild cognitive impairment (MCI). Statistical analysis showed significant differences (*p* < 0.05) between tortuosity indices determined for healthy controls (HC) vs. MCI and HC vs. AD in most of the analyzed structures. Other clinically used BMs have also been incorporated in the analysis: beta-amyloid and tau protein CSF and plasma concentrations, as well as other image-extracted parameters. A classification strategy using random forest (RF) algorithms was implemented to discriminate between three samples of the studied populations, selected from the ADNI database. Classification rates considering only image-extracted parameters show an increase of 9.17%, when tortuosity is incorporated. An enhancement of 1.67% is obtained when BMs measured from several modalities are combined with tortuosity.

## 1. Introduction

Alzheimer's disease (AD) is a neurodegenerative disorder that leads to irreversible gradual loss of cognitive and intellectual capabilities, thus limiting a subject's performance in daily activities. It has been reported that this illness gradually provokes changes in brain morphology, leading to loss of cognitive activities such as memory, orientation, and language. It is related to aging and represents between 50% and 75% of all cases of dementia in global population [1]. The clinical criteria of the National Institute on Aging-Alzheimer's Association (NIA-AA) generally recognize three phases principal of the progression of AD [2]: preclinical healthy stage, mild cognitive impairment (MCI), and dementia due to AD.

The main objective of the diagnostic criteria is to support experts in the early identification and treatment of AD [2]. In this context, different biomarkers (BM) and surrogate biomarkers have been considered in the diagnosis:

- Cognitive tests, the most clinically used being the Mini-Mental State Examination (MMSE) and the Clinical Dementia Rating (CDR). These neuropsychological parameters, together with other clinical examinations, are the most frequently used in clinical practice [2].
- Biochemical markers related to the concentration of beta-amyloid peptides (A*β*40 and A*β*42) or tau protein. Some authors have studied the association between AD and A*β*40 and A*β*42 plasma levels, with inconclusive result [3] while others have demonstrated that the A*β*42/A*β*40 ratio can predict the development of the disease, especially if it is measured in total blood, not only in plasma [4].
- Image-extracted biomarkers. Recently, magnetic resonance imaging (MRI) emerged as a noninvasive clinical technique to support the medical diagnosis of AD [2, 5, 6]. Specifically, brain parameters extracted from MRI, such as volume of different brain structures, atrophy of the hippocampus, and cortical thickness, are the most common in clinical diagnosis. However, clinical evaluation of neuroimages is mainly based on visual inspection by the neuroradiologists, who are not always able to detect early signs of neurodegeneration from a qualitative observation of brain structures [6].

A recent interest has arisen on the medical and neuroimaging communities to incorporate techniques taken from artificial intelligence and machine learning fields for AD diagnosis. Also, new image-extracted biomarkers have appeared for the early detection of brain changes. Many studies focus on combining BMs of different kinds and applying pattern recognition techniques to identify early symptoms of the disease, especially during the MCI stage [6].

Some research studies recently reported on the classification of patients with AD in its different phases and described the use of biomarkers and pattern analysis to solve the problem of early detection of symptoms in subjects with MCI. Janousova et al. [7] reported the analysis of four populations: HC; progressive MCI that converts to AD (MCIc); stable nonconverting MCI (MCInc); and AD. They considered fluorodeoxyglucose (FDG) levels obtained from PET images and intensities obtained from T1-weighted MRI as features for classification employing a support vector machine (SVM). The authors describe a computationally efficient procedure, based on a combination of penalized regression (regression using the elastic net penalty) and a resampling method, for the identification of localized brain regions that are highly discriminative between two groups of MR images. Perez-Gonzalez et al. [8] report the analysis and SVM classification of HC, MCI, and AD populations. They propose a new feature based on discrete compactness that is combined with absolute and normalized volumes for WM and GM (global and by brain lobes).

Gray et al. [9] report a study where four populations (HC, MCIc, MCInc, and AD) are analyzed. A random forest (RF) sorter with 5000 trees was fed with a feature vector including volumetric measures, FDG-PET voxel intensities of the whole brain, CSF-derived measures, and genetic information. The authors report a classification strategy based on pairwise similarity measures derived from random forest classifiers.

Payan et al. [10] use a 3D convolutional neural classifier networks to identify HC, MCI, and AD populations. The proposed algorithm uses the whole information of MRI images as input data. The results are contrasted versus a 2D neural network classifier, obtaining an increase of 3.94% in accuracy. Lebedev and colleagues [11] conducted a study where two populations (AD versus HC) were analyzed, using a 1000-tree RF classifier. The used features were noncortical volumes, cortical thickness, Jacobian maps, and sulcal depth. In this research, the authors propose an analysis of different MRI metrics using random forest classifiers in a cohort study. The authors state that they obtain better classification rates than other models such as SVM with linear kernel.

Sivapriya et al. [12] reported a research with three studied populations (AD, MCI, and HC). This study proposes an ensemble feature selection approach using different classifiers with a particle swarm optimization search strategy and the merit merge technique. The features considered in the RF classification process were volumetric measures, FDG-PET, and neuropsychological scores.

Recently, Dimitriadis et al. [13] explored the efficacy of a novel scheme that includes multiple feature selections (MRI-based features) via RF. This research considered four classes (HC, MCIc, MCInc, and AD). This method includes subsets of the whole set of features per lobules and hemispheres. The final decision of the classification uses a fusion approach of different methods and the ensemble classification via majority voting. Ramirez et al. [14] proposed a system based on feature standardization, ANOVA feature selection, partial least squares feature dimension reduction, and an ensemble of one vs. rest random forest classifiers. The system was trained and evaluated on ADNI datasets that consist of T1-weighted MRI morphological measurements from HC, MCInc, MCIc, and AD subjects. In other research, Salvatore et al. [6] propose a machine learning classifier for the automatic early diagnosis and prognosis of AD by means of features extracted, selected, and optimized from structural MRI brain images. A linear SVM was used to separate groups of subjects and was designed to perform multilabel automatic classification into the following four classes: HC, MCInc, MCIc, and AD.

Other authors have reported cortical thickness as an effective biomarker to distinguish between control subjects and patients with AD. Lerch et al. [15] performed a study in which the cortical thickness of different cerebral structures was automatically measured, using three classification methods: a linear discriminant (LDA), a quadratic discriminant (QLA), and a logistic regression model to discriminate between populations of healthy controls versus patients with AD. Du et al. have also reported the use of cortical thickness as a useful substitute biomarker to distinguish between frontotemporal dementia and AD. They carried out a study where controls, patients with frontotemporal dementia, and patients with Alzheimer's disease were analyzed [16]. A logistic regression was used as classification model, and cortical thickness and volume were used as biomarkers.

Considering these previous studies, it can be noticed that there is an active field of research in the study of different image biomarkers that can provide complementary information for support in the early detection of AD. Also, it has been possible to corroborate that the integration of multimodal studies can provide a better performance in the discrimination of the different stages of Alzheimer's disease. For these reasons, in this work, the three-dimensional tortuosity measured in several cortical regions segmented from T1-weighted MR images is proposed as a novel biomarker. It is intended to be used as a complementary parameter that quantifies structural changes. Although tortuosity has been proposed as a morphological BM for different medical applications [17–19], this indicator has not yet been evaluated in support of the early diagnosis of AD. Also, a random forest (RF) classifier is proposed and designed to perform multilabel automatic classification, using multimodal features, into the following three subject classes: HC, MCI, and AD; in this case, the MCI population corresponds to stable MCInc subjects that did not evolve to Alzheimer's dementia.

## 2. Materials and Methods

In this section, the proposed methodology is presented, starting with dataset description, as well as the inclusion criteria of patients with AD, MCI, and HC. Subsequently, the segmentation process of white matter, gray matter, and cerebrospinal fluid using Statistical Parametric Mapping is presented. Also, image-based biomarkers are explained in detail (absolute and normalized volume, cortical thickness, discrete compactness, and tortuosity), as well as the statistical tests applied to evaluate their discriminant capability. Finally, a classification scheme based on random forest is presented in which image-based, biochemical, and surrogate cognitive biomarkers are integrated. As described above, the aim is to discriminate between AD patients, MCI subjects, and healthy controls, in order to observe the classification power of the tortuosity index when it is combined with other biomarkers of different modalities.

### 2.1. Datasets

Data used in the preparation of this article were obtained from the Alzheimer's Disease Neuroimaging Initiative (ADNI) database (adni.loni.usc.edu). The ADNI was launched in 2003 as a public-private partnership, led by Principal Investigator Michael W. Weiner, MD. The primary goal of the ADNI has been to test whether serial magnetic resonance imaging (MRI), positron emission tomography (PET), other biological markers, and clinical and neuropsychological assessment can be combined to measure the progression of mild cognitive impairment (MCI) and early Alzheimer's disease (AD).

Population assignment was carried out as reported in [20] by ADNI experts with the following inclusion criteria for HC: MMSE scores between 24 and 30; CDR of zero; and absence of depression, MCI, and dementia. Inclusion criteria for MCI were as follows: MMSE scores between 24 and 30; CDR of 0.5; objective memory loss, measured by education-adjusted scores on Wechsler Memory Scale Logical Memory II; absence of significant levels of impairment in other cognitive domains; and absence of dementia. Inclusion criteria for AD were as follows: MMSE scores between 20 and 26 and CDR of 0.5 or 1.0. Detailed description of inclusion/exclusion criteria can be found in the ADNI protocol (http://www.adni-info.org/Scientists/ADNIStudyProcedures.aspx).

The three populations selected for this research were constituted considering, in addition to the abovementioned criteria, the completeness of the following information:

- Tau protein and A*β*42 levels, measured in cerebrospinal fluid (for approximately 20% of each population this information is provided [20])
- A*β*40 and A*β*42 levels obtained from blood plasma
- MR volumes acquired in similar conditions (T1-weighted, at 1.5 T, voxel resolution of 1.2 × 0.93 × 0.93 mm^3^)
- Cognitive scores evaluated by MMSE and CDR tests
- Groups paired by age
- All biomarkers recorded in nearby dates

#### 2.1.1. Training/Validation Dataset

Considering these inclusion criteria, the training populations consisted of 40 healthy controls (HC), 40 subjects with stable mild cognitive impairment (MCI), and 40 patients with Alzheimer's disease (AD). The corresponding demographic and neuropsychological scores are shown in Table 1. This set was used for statistical analysis and to train and design the classification strategy.

#### 2.1.2. Holdout Blind Test Set

The same inclusion criteria were considered to constitute a blind dataset composed of 10 HC, 10 MCI, and 10 AD. The corresponding demographic information is shown in Table 2. This dataset was never seen during training step and was exclusively used for final testing.

### 2.2. MR Image Processing

As a first step, global white and gray matters were segmented from brain MR volumes; the latter was also subdivided into several substructures, to separate parietal and temporal lobes, for both hemispheres of the 150 subjects. To this purpose, the “Statistical Parametric Mapping” (SPM) [21] tool was used, with the following sequence: volume orientation, image realignment and registration, and segmentation. In the first step, the corpus callosum was used as anatomic reference for volume orientation; this structure is frequently used for this purpose because it is easily identified in most subjects.

In order to obtain parietal and temporal lobes, the Individual Brain Atlas using Statistical Parametric Mapping (IBASPM) was used [22]; this is an available toolbox for SPM platform, widely employed in neuroimage processing. Lobe parcellation is carried out as follows:

- Segmentation This process must be previously carried out with the SPM platform to separate cerebrospinal fluid (CSF), gray matter (GM), and white matter (WM) in the native space of the original images.
- Normalization T1-image is assigned to the stereotactic MNI space, using the ICBM 152 T1 mask. This step provides a spatial transformation matrix that will be subsequently used.
- Labelling White matter voxels that were identified in the segmentation step are mapped to the inverse transformation matrix produced during normalization. Every voxel is assigned to a unique structure that is contained in the atlas space. The final volume labels are applied to subcortical structures according to previous probabilities assigned by the atlas.
- Individual Atlas Deformation fields calculated during the normalization step are inverted, remaining holes are filled, and isolated dots are eliminated to obtain the final structure. With this last step, an individual atlas parceled by structures is obtained. From this procedure, six brain structures were obtained: GM, WM, right and left temporal lobes (RTL and LTL), and right and left parietal lobes (RPL and LPL).

### 2.3. Volume (V) and Normalized Volume (NV)

As a first step, an interpolation of MR volumes using B-spline functions was applied, in order to have measures with isometric resolution of 1 mm^3^. After segmentation, the volume is easily estimated by counting the number of voxels (*n*) that belong to regions of interest. Normalization of these volumes was carried out by dividing the total number of voxels of a given structure by the intracranial volume, which is computed by adding those voxels corresponding to GM, WM, and CSF.

### 2.4. Cortical Thickness (CT)

Cortical thickness measurements were made using FreeSurfer version 6.0 which is documented and freely available for download online (http://surfer.nmr.mgh.harvard.edu/). The technical details of these procedures are described in previous publications [23–26]. This processing includes the following:

- Motion correction and averaging of multiple volumetric T1-weighted images [26]
- Removal of non-brain tissue using a hybrid watershed/surface deformation procedure, automated Talairach transformation, and segmentation of the subcortical white matter and deep gray matter volumetric structures [25]
- Gray matter and white matter boundary and automated topology correction [24]
- Surface deformation following intensity gradients to optimally place the gray/white and gray/cerebrospinal fluid borders at the location where the greatest shift in intensity defines the transition to the other tissue class [23]

Procedures for the measurement of cortical thickness have been validated against histological analysis [27] and manual measurements [28].

In this work, we calculated the mean global cortical thickness and the temporal and superior parietal lobe cortical thicknesses. For all these structures, right and left hemispheres were analyzed to be included as biomarkers in the classification process.

### 2.5. Discrete Compactness (DC)

This metric has been reported as a useful biomarker for the classification of HC, MCI, and AD [8]. DC is computationally simple to calculate, and it varies in a range of zero (null compactness) and one (totally compact voxels). It is also invariant to rotation, scaling, or translation, qualities that are useful to eliminate bias in the measurement. It can be expressed for a 3D object as the ratio between the area of its enclosing surface and its volume as follows: (area)^3^/(volume)^2^ and relates the number of voxels (*n*) and the enclosing area (*A*) by the following mathematical expression [29]:

(1) $$\begin{array}{c} \text{DC}=\frac{n-\left(A/6\right)}{n-{\left(\sqrt[{3}]{n}\right)}^{2}}. \end{array}$$

### 2.6. Tortuosity (TR)

Tortuosity is an intrinsic property possessed by curved surfaces. This characteristic is one of the most important measures of shape used to calculate deformations in different objects, which allows to quantify the degree of turns or detours of a surface [30].

Different diseases such as hypertension and vasculopathies can affect the morphology of blood vessels, so that tortuosity measures have been proposed in the literature to quantify the morphological changes associated with these pathological processes [17, 19]. For these reasons, we consider that tortuosity can be a useful metric to determine the deformation of brain structures when they are affected by Alzheimer's disease.

Normalized tortuosity has been reported by Bullitt et al. [17] and has been estimated by the sum of angles metric (SOAM) method. The procedure to determine the 3D angle between consecutive 3D triads is as follows [17]:

(i) A mesh is obtained from the 3D skeletonization process, constituting a set of *N* ordered points *P*_*k*_, with coordinates (*x*_*k*_, *y*_*k*_, *z*_*k*_)
(ii) Vectors *X*, *Y*, and *Z* are defined, containing, respectively, the set of *x*_*k*_, *y*_*k*_, and *z*_*k*_ coordinates of the array of points
(iii) From these vectors, the following two-column, *k*-row arrays are conformed: T1 = [*Y X*], T2 = [*Z Y*], and T3 = [*Z X*]
(iv) Each array is normalized
(v) The following elements are computed:
  (2) $$\begin{array}{c} \text{Plane angle}:\;{\text{IP}}_{k}=\cos^{-1}\left({\left(\frac{T1}{\left|T1\right|}\right)}^{{}^{\circ}}\left(\frac{T2}{\left|T2\right|}\right)\right),\\ \text{Torsion angle}:\;{\text{TP}}_{k}=\cos^{-1}\left({\left(\frac{T1\times T2}{\left|T1\times T2\right|}\right)}^{{}^{\circ}}\left(\frac{T2\times T3}{\left|T2\times T3\right|}\right)\right),\\ \text{Total angle}:\;{\text{CP}}_{k}=\sqrt{\left(IP_{k}\times IP_{k}\right)+\left(TP_{k}\times TP_{k}\right)},\\ \text{SOAM}:\text{ TR}=\frac{\left(\sum_{k=1}^{N-3}CP_{k}\right)}{\left(\sum_{k=1}^{N-1}\left|P_{k}-P_{k-1}\right|\right)}. \end{array}$$

In this work, the torsion angle is not considered for SOAM computation because, as Bullitt reported previously for vessel shapes measured from angiography images, it does not contribute for healthy and sick subjects' comparison and only adds noise [31].

### 2.7. Statistical Analysis

After calculating the biomarkers proposed in previous sections, a statistical analysis was carried out on the training dataset (*N* = 120) to find between-classes and between-biomarkers significant differences. We applied a Kruskal–Wallis test (*p* ≤ 0.05) between the three classes, since the calculated metrics do not follow a normal distribution. Afterwards, a Mann–Whitney–Wilcoxon *post hoc* analysis was carried out to identify between-class differences.

### 2.8. Classification Scheme

To classify the studied populations into three groups, a random forest (RF) was used. Lebedev et al. have reported that RF performance is superior to SVM's for this application [11]. It initially received 35 features:

- Image-extracted BM: V, NV, DC, CT, and TR for the six brain structures (GM, WM, RTL, LTL, RPL, and LPL), totaling 30 features
- Biochemical markers: tau protein and A*β*42 CSF levels; A*β*40 and A*β*42 obtained from blood plasma; and A*β*40/A*β*42 ratio (5 features)
- Surrogate cognitive biomarkers: CDR and MMSE. They were considered as the gold standard, against which the results obtained in the classification process were compared

Random forests are defined as a collection of classifiers structured as a tree, denoted by *f*(*x*, *θ*_*k*_), where *x* is the vector of the input class and *θ*_*k*_ is a vector of random data independently distributed [11]. For the training feature vector *θ*_*k*_ of length *n*, new subsets are parcelled into *m* partitions of length *n*1 (*n*1<*n*) by sampling and replacement. The obtained *m* models are combined for training and classification of the random forest [14].

Feature selection and importance assignment for each variable are implicitly done during RF classification with the Gini impurity index, which is a nonparametric measure of a feature's prediction power. For each binary node *n* in the decision tree, the Gini index is calculated as follows [32]:

(3) $$\begin{array}{c} \text{Gini}\left(n\right)=1-\overset{2}{\underset{j=1}{\sum }}p_{j}^{2}, \end{array}$$

where *p*_*j*_ is the relative frequency of class *j* in the node *n*. The RF was designed with the following parameters: 150 trees in the forest; maximum depth of the tree of 8; and minimum number of samples required to divide an internal node of 2. The classifier generates three possible outputs: HC, MCI subjects, and patients with AD.

All RF-based classifiers were designed following a 10-fold cross-validation, using the training dataset (*N* = 120, 40 subjects of each population) described in Section 2.1.1, and therefore, accuracies are reported as central and dispersion measures obtained during training. Afterwards, for final validation, the confusion matrix and performance of the classifier were calculated on the holdout blind dataset (*N* = 30, 10 subjects for each class).

To evaluate the contribution of tortuosity as a structural biomarker of Alzheimer's disease, several feature combinations were tested, first considering only image-extracted biomarkers and then incorporating biochemical and cognitive parameters. All combinations were tested with and without tortuosity.

## 3. Results

### 3.1. Segmentation

**Figure 1:**
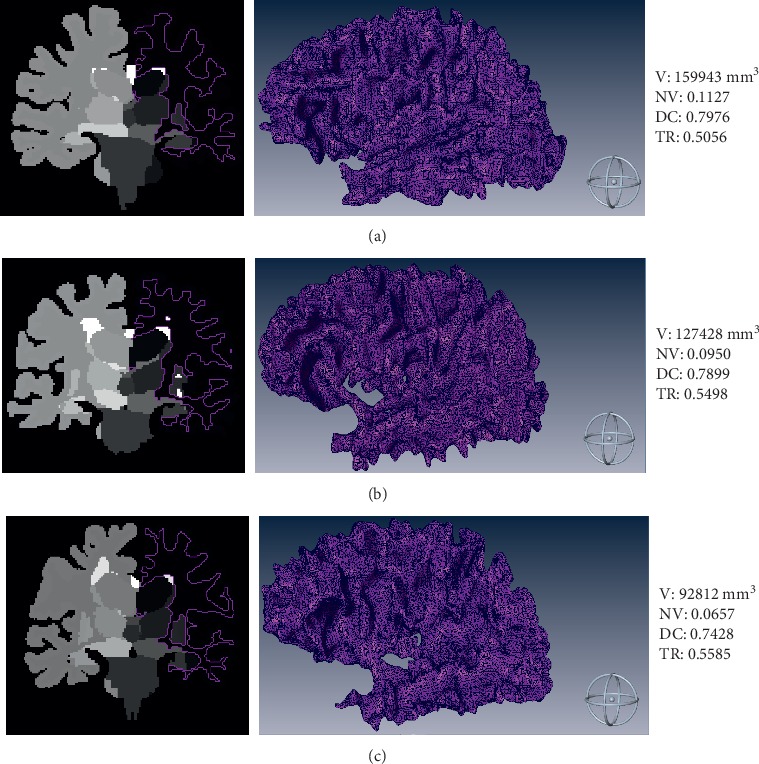
Axial slices of left hemisphere white matter obtained after the segmentation process: (a) healthy control, (b) subject with MCI, and (c) patient with AD. The three-dimensional reconstructions for each of the structures are observed, as well as the corresponding metrics of V, NV, DC, and TR.

Figure 1 shows an example of segmentation and labelling of brain structures for three subjects HC, MCI, and AD, respectively. The contoured regions correspond to white matter in the left hemisphere. The decrease in volume and normalized volume that occurs between healthy subjects and patients with AD can be observed. Discrete compactness also decreases for this structure and tortuosity increases as the pathological process advances. The metrics calculated for this structure are indicated in the figure.

### 3.2. Statistical Analysis

**Figure 2:**
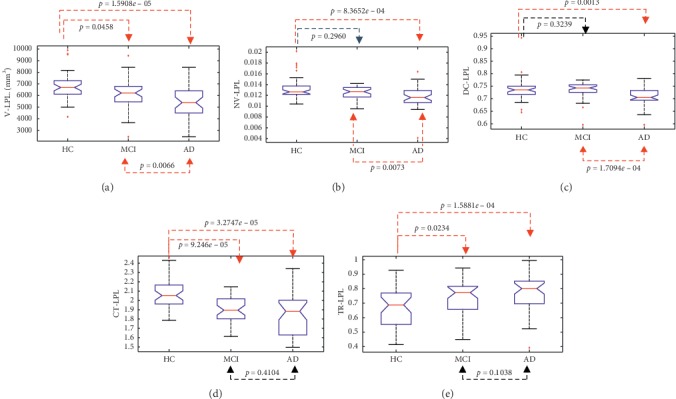
Statistical analysis for volume (a), normalized volume (b), compactness (c), cortical thickness, (d) and tortuosity (e) measured in left parietal lobe for HC, MCI, and AD populations in that order. Significant differences (*p* < 0.05) are highlighted in red.

Absolute volume, normalized volume, discrete compactness, cortical thickness, and tortuosity were computed for all the segmented brain structures of the training dataset. As an example, Figure 2 shows the statistical analysis carried out in the left parietal lobe for the three populations; significant differences are highlighted in red, for *p* < 0.05.

The same statistical analysis was applied to all segmented structures (Table 3), as well as to cognitive and biochemical biomarkers; as described in Section 2.1 of the latter, only BM measured in CSF showed significant differences between classes, and therefore, they were removed from subsequent analysis. Although the ADNI dataset is classified according to MMSE [20], this cognitive score is widely used for clinical diagnosis, and several authors have included it for subsequent assignment in machine learning medical applications. Thus, we have considered the MMSE score as surrogate biomarker, to be combined with the other image and biochemical BMs to constitute the multimodal feature vector.

### 3.3. Random Forest Classification

As explained in previous sections, 120 subjects of the three analyzed classes were used (40 HC, 40 MCI, and 40 AD) to train the RF classifier. Several BM combinations were tested, in order to determine the contribution of each modality, as shown in Table 4. The corresponding accuracies are reported as central and dispersion measures obtained during cross-validation.

Generalization capability for the proposed strategy was determined as the accuracy measured in the holdout test set (Table 5), which constituted 30 cases (10 HC, 10 MCI, and 10 AD) not previously seen by the classifier.

The first two classifications were carried out including only image-extracted BMs, to consider the individual effect of tortuosity (2^nd^ row), compared to previously reported indices (1^st^ row). CSF BMs were incorporated in the feature vector, without (3^rd^ row) and with (4^th^ row) tortuosity. All modalities including MMSE as surrogate biomarker have been considered in the last two rows, again with (6^th^ row) and without (5^th^ row) tortuosity.

**Figure 3:**
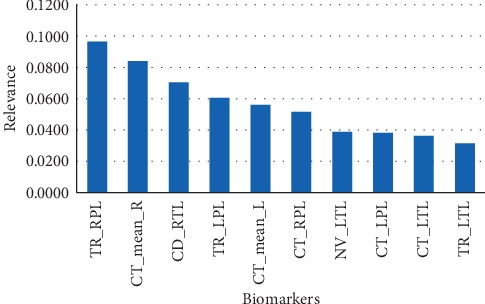
Feature relevance during RF classifier training and design.

To identify their contribution, each feature is ranked according to the relevance measured by the Gini index in the RF classification.  Figure 3 shows the ten most important features determined in this process. In particular, tortuosity measured at the parietal lobe together with the average cortical thickness contributes the most to the final classification.

In order to identify the error type introduced by this process, confusion matrices were obtained for all combinations, which are shown in Table 6.

## 4. Discussion

### 4.1. Statistical Analysis

It can be observed in Figures 2(a) and 2(b) that brain volumes decrease for this structure: absolute volume showed a statistical significance *p*=4.3374*e* − 05 between the three classes, while for normalized volume it was *p*=0.0015. These results concur with those reported by Karas et al. [33] and are probably due to morphological changes associated with AD evolution. It can also be seen that discrete compactness (Figure 2(c)) presents a similar decreasing behavior because it is related to structure density that is affected during AD progression; a statistical significance *p*=1.9878*e* − 04 was obtained for the difference between the three populations. These results are in agreement with those reported in [8], showing that compactness is a helpful index for early screening of changes associated with MCI and AD. Morphological changes are associated with structural atrophy in some brain areas, appearing as a result of neuronal and tissue loss, typical of neurodegenerative diseases [33].

Figure 2(d) presents the behavior of cortical thickness, which decreases according to the progression of the disease and shows statistical differences (*p* < 0.001) between HC and MCI populations and between HC subjects versus patients with AD. Similar results have been reported in [34] between control subjects and subjects with typical Alzheimer's disease (tAD) where it is shown that the cortical thickness is thinner in subjects with tAD in regions of the temporal and parietal lobes.

In contrast, tortuosity shows a growing tendency (Figure 2(e)), possibly associated with the erosion of brain structures and with the curvature increase of cortical circumvolutions, appearing as AD progresses. Significant differences are observed between the three classes (*p*=6.09*e* − 04).

Results shown in Table 3 indicate that tortuosity determined for all brain structures is significantly different between HC vs. MCI (excepting right temporal lobe) and between HC vs. AD (excepting left temporal lobe). The former comparison (HC vs. MCI) must be highlighted because it has more clinical relevance, when subjects are beginning to present precocious symptoms of the disease. In this case, the tortuosity metric is the one that most frequently appears with statistical differences in all the analyzed brain structures, except for the right temporal lobe, while cortical thickness, which is widely used in the literature, showed differences in four out of six brain structures. In the comparison of HC vs. AD, all biomarkers are sensible to atrophic changes that are already evident at this stage of the disease, and therefore, they present significant differences for almost all brain structures. Finally, between the MCI and AD classes, tortuosity and cortical thickness are the least relevant biomarkers because the former only presents statistical differences in the right temporal lobe, while the latter is not significantly distinguishable in any of the structures. To separate these classes, other biomarkers such as volume and discrete compactness can be used. With these comparisons, it can be corroborated that the combination of several image biomarkers in the multivariate analysis can provide additional complementary information, in contrast to the use of biomarkers individually.

Statistical analyses applied to cognitive and biochemical parameters indicate that those indices presenting significant differences are cognitive tests (MMSE and CDR) and CSF proteins (tau and beta-amyloid); these results are coincident with those reported in [20] for populations extracted from the same ADNI database. In any case, there were no significant differences for indices obtained from blood plasma, probably due to a weak association between these parameters and the disease, as reported in [35]. The authors suggest that plasma A*β* increase is not necessarily associated with an overproduction of amyloid on the brain, but with renal excretion reduction or peripheral vessel disorders, and therefore presents a weak association with AD [35]. For this reason, biochemical markers considered for classification include only CSF levels.

### 4.2. Random Forest Classification

It can be observed in Table 4 that TR contributes by itself to increase accuracy more than 9% considering only image BMs (rows 1 and 2). Combining image with biochemical BMs, a 2.5% increase can be seen in rows 3 and 4, when adding TR in the classification. Finally, when incorporating all image, biochemical, and cognitive indicators, it can be seen that TR contributes by 1.67% to accuracy (rows 5 and 6). These results reflect that TR contributes to the classification in the three scenarios: using only image BMs, image plus biochemical, and in the incorporation of all features, in which a 90% of accuracy was obtained with the training dataset (6th row last column).

Results obtained with the test dataset (Table 5) show that tortuosity contributed with an increase of up to 6.66% when using only image-extracted BMS, of 6.67% when adding CSF levels, and of 3.33% when considering all biomarkers.

These results are comparable with those obtained by Sivapriya et al. [12] that report an accuracy of 96.3% during 5-fold cross-validation of an RF, but considering parameters extracted from PET images. Our results can also be contrasted with performances reported by Salvatore et al. [6] that show classification rates of 59% (HC vs. MCI), 90% (MCI vs. AD), and 96% (HC vs. AD), considering image-extracted BM as well as cognitive scores. It must be noted that in, all these studies, the incorporation of neuropsychological tests considerably increases classification rates because cognitive changes are a clear expression of AD progression and are therefore employed as a primary clinical criterion for its diagnosis.

Lerch et al. [15] report a study where several brain structures are analyzed, incorporating the average cortical thickness of the entire cortex to discriminate groups of patients and controls. Their results show a precision of 75%, a sensitivity of 79%, and a specificity of 71%, using quadratic discriminant analysis. The authors report the highest accuracy (94%) when parahippocampal gyration is included. However, it should be noted that these results only consider two populations (healthy controls versus AD patients) with only 17 and 19 subjects, respectively [15].

The results reported in [16] suggest that the cortex' thickness in patients with Alzheimer's disease was thinner (*p* < 0.001) in the bilateral parietal region, compared to patients with frontotemporal dementia. Compared to normal subjects, patients with Alzheimer's disease had a thinner cortex mainly in the bilateral, frontal, parietal, temporal, and occipital lobes (*p* < 0.001). The analyzed population was 23 control subjects, 22 patients diagnosed with AD, and 19 patients with frontotemporal dementia. Classification accuracy for this study was 96% ± 3% using cross-validation for differences between control subjects versus AD patients. To differentiate patients with frontotemporal dementia, the best result taking into account cortical thickness was 85% ± 6%. To differentiate between the two types of dementia, the result was 82% ± 1% [16].

Nanni et al. [36] report classification rates between 85.5% and 92.4%, when comparing pairs of groups (MCI vs. HC and AD vs. HC), but employing a large number of image features (2000) selected with several methods. Perez-Gonzalez et al. [8] also test the efficiency of discrete compactness for the classification of populations' pairs obtaining accuracies going from 85% (MCI vs. HC) to 98.3% (AD vs. HC). In contrast, Dimitriadis et al. [13] report a 61.9% accuracy in the classification of four groups (HC, MCIc, MCInc, and AD), but it must be noted that discriminating between MCIc and MCInc classes can cause the apparently low performance of their methods. Payan et al. [10] also carry out a multigroup classification (HC, MCI, and AD) leading to a 89.5% accuracy, but through the use of a complex method based on 3D convolutional neural networks.

Results displayed in Table 6 show that the greater errors appear in the MCI group assignment that our method erroneously classifies as AD and in some cases to the HC class. This contrasts with results reported by Dimitriadis et al. [13] and by Ramirez et al. [14] that misclassify a 35% and 40%, respectively, of MCI cases to the HC class, which can lead to an incorrect clinical management of those subjects. On the other hand, the combination (V, NV, DC, CT, TR, CSF, and MMSE) tested in this research attains a 90% classification of HC and AD populations and 80% classification of HC and MCI populations, in contrast to 60% and 62.5% for the HC class and to 95% and 92.5% for the AD class, respectively, obtained by the same cited authors [13, 14]. It is necessary to mention that they include the MCIc class that can cause a decrease in the overall performance of their methods.

The previous results suggest that tortuosity is a metric that can provide relevant information in the early diagnosis of AD. This is based on two fundamental arguments. The first is the statistical results shown in Table 3, where the contribution of this biomarker to discriminate between the classes of control subjects versus subjects with MCI (stage prior to the AD) is appreciated. The second argument is related to the classification process shown in Table 5, where it can be observed that in all cases when tortuosity was included as a feature, the classification accuracy increased. For these reasons, we think that this indicator has an excellent potential to become an effective image biomarker in the early diagnosis of AD.

## 5. Conclusions

In this work, a classification strategy that combines biomarkers obtained from several modalities for early AD detection was presented. The main contribution is the introduction of tortuosity measures, computed through SOAM, as a promising morphological parameter to be considered in early stages of the disease. Results show that tortuosity is sensitive to morphology changes appearing in several brain structures, whose atrophic deterioration can be detected as early as the MCI stage using the combination of biomarkers proposed in this paper.

Results obtained in this study show significant differences of tortuosity indices for most of the analyzed structures, allowing the discrimination between HC vs. MCIs and HC vs. AD; these findings have special clinical relevance for the detection of AD's early symptoms. Nevertheless, although tortuosity presented a better discrimination capability than other image-extracted parameters (volume, discrete compactness, and cortical thickness) to separate HC from the other two groups, it did not show the same performance to distinguish between MCI and AD subjects. In this case, other features were more efficient than tortuosity, which corroborates that the integration and adequate selection of different types of BMs provide higher accuracies.

The proposed classification strategy incorporates multimodal information and allows attaining an accuracy of 86.66% during the test stage with previously unseen data. These findings suggest that tortuosity can be considered as an image-extracted BM, helpful in the detection of early stages of AD when subjects present the first clinical symptoms of the disease.

## Figures and Tables

**Table 1:** Training/validation dataset: demographic data and MMSE and CDR scores for subjects selected from the ADNI database.

	HC (*N* = 40)	MCI (*N* = 40)	AD (*N* = 40)
Age (years) (*μ* ± *σ*)	76.2 ± 4.5	74.9 ± 7.2	76.9 ± 5.0
Gender (F/M)	22/18	18/22	21/19
MMSE (rank/*μ* ± *σ*)	28–30/29.4 ± 0.74	22–29/26.2 ± 1.7	12–27/21.1 ± 3.3
CDR (sum of boxes) (rank/*μ* ± *σ*)	0	0.5–4 (2.1 ± 0.3)	2.5–13 (6.4 ± 2.3)

**Table 2:** Holdout test dataset: demographic data and MMSE and CDR scores for subjects selected from the ADNI database.

	HC (*N* = 10)	MCI (*N* = 10)	AD (*N* = 10)
Age (years) (*μ* ± *σ*)	75.2 ± 3.7	72.1 ± 4.7	73.6 ± 8.0
Gender (F/M)	4/6	2/8	5/5
MMSE (rank/*μ* ± *σ*)	29–30/29.6 ± 0.5	24–28/26.0 ± 1.3	15–25/20.1 ± 4.6
CDR (sum of boxes) (rank/*μ* ± *σ*)	0	1–3.5 (2.2 ± 0.8)	5.5–12 (7.1 ± 2.7)

**Table 3:** Statistical comparison for morphological imaging BMs. Only differences with a significance value *p* < 0.05 are marked with ^*∗*^.

	HC vs. MCI					HC vs. AD					MCI vs. AD				
	V	NV	DC	CT	TR	V	NV	DC	CT	TR	V	NV	DC	CT	TR
Global gray matter	^*∗*^	^*∗*^		^*∗*^	^*∗*^	^*∗*^	^*∗*^		^*∗*^	^*∗*^					
Global white matter			^*∗*^		^*∗*^	^*∗*^	^*∗*^	^*∗*^		^*∗*^	^*∗*^	^*∗*^			
Right temporal lobe						^*∗*^	^*∗*^	^*∗*^	^*∗*^	^*∗*^	^*∗*^	^*∗*^	^*∗*^		^*∗*^
Left temporal lobe		^*∗*^		^*∗*^	^*∗*^		^*∗*^	^*∗*^	^*∗*^		^*∗*^		^*∗*^		
Right parietal lobe	^*∗*^	^*∗*^	^*∗*^	^*∗*^	^*∗*^	^*∗*^	^*∗*^	^*∗*^	^*∗*^	^*∗*^	^*∗*^		^*∗*^		
Left parietal lobe	^*∗*^			^*∗*^	^*∗*^	^*∗*^	^*∗*^	^*∗*^	^*∗*^	^*∗*^	^*∗*^	^*∗*^	^*∗*^		

**Table 4:** Performance of the RF classifier on the training/validation dataset, for different combinations of biomarkers used as feature vector.

	Biomarker							
	V, NV	DC	CT	TR	Biochemical (CSF)	Cognitive (MMSE)	Number of features	Validation accuracy (*N* = 120) (*μ* ± *σ*)
1	✓	✓	✓				24	67.50 ± 0.16%
2	✓	✓	✓	✓			30	76.67 ± 0.33%
3	✓	✓	✓		✓		26	78.33 ± 0.23%
4	✓	✓	✓	✓	✓		32	80.83 ± 0.27%
5	✓	✓	✓		✓	✓	27	88.33 ± 0.11%
6	✓	✓	✓	✓	✓	✓	33	90.00 ± 0.12%

**Table 5:** Performance on the holdout test dataset, for different combinations of biomarkers used as feature vector.

	Biomarker							
	V, NV	DC	CT	TR	Biochemical (CSF)	Cognitive (MMSE)	Number of features	Accuracy test set (*N* = 30) (%)
1	✓	✓	✓				24	60.0
2	✓	✓	✓	✓			30	66.66
3	✓	✓	✓		✓		26	63.33
4	✓	✓	✓	✓	✓		32	70.0
5	✓	✓	✓		✓	✓	27	83.33
6	✓	✓	✓	✓	✓	✓	33	86.66

**Table 6:** Confusion matrices for different biomarkers' combinations.

BM: V, NV, DC, CT			
Accuracy: 60%			
	HC	MCI	AD
HC	8	2	0
MCI	0	4	6
AD	0	4	6

BM: V, NV, DC, CT, TR			
Accuracy: 66.66%			
	HC	MCI	AD
HC	9	1	0
MCI	0	4	6
AD	0	3	7

BM: V, NV, DC, CT, CSF			
Accuracy: 63.33%			
	HC	MCI	AD
HC	7	3	0
MCI	1	5	4
AD	1	2	7

BM: V, NV, DC, CT, CSF, TR			
Accuracy: 70%			
	HC	MCI	AD
HC	7	3	0
MCI	1	7	2
AD	1	2	7

BM: V, NV, DC, CT, CSF, MMSE			
Accuracy: 83.33%			
	HC	MCI	AD
HC	10	0	0
MCI	1	7	2
AD	0	2	8

BM: V, NV, DC, CT, CSF, MMSE, TR			
Accuracy: 86.66%			
	HC	MCI	AD
HC	10	0	0
MCI	0	8	2
AD	0	2	8

## Data Availability

The neuropsychological assessment, biological markers, and MRI data used to support the findings of this study were supplied by the Alzheimer's Disease Neuroimaging Initiative (ADNI) database, available at adni.loni.usc.edu. This research has the Data and Publications Committee (ADNIDPC) approval to process these data, which were correctly used and referenced in the research worK. Data used in preparation of this article were obtained from the Alzheimer's Disease Neuroimaging Initiative (ADNI) database (adni.loni.usc.edu). As such, the investigators within the ADNI contributed to the design and implementation of ADNI and/or provided data but did not participate in analysis or writing of this report. A complete listing of ADNI investigators can be found at http://adni.loni.usc.edu/wpcontent/uploads/how_to_apply/ADNI_Acknowledgement_List.pdf.
